# Comorbidities, Treatment and Survival Rates of Chronic Thromboembolic Pulmonary Hypertension in a Regional Centre

**DOI:** 10.3390/jcm13102754

**Published:** 2024-05-07

**Authors:** Razvan Adrian Bertici, Nicoleta Sorina Bertici, Amalia Ridichie, Ovidiu Fira-Mladinescu

**Affiliations:** 1 Department XIII Pulmonology, Victor Babes University of Medicine and Pharmacy Timisoara, Eftimie Murgu Sq. No. 2, 300041 Timisoara, Romania; razvan.bertici.umfvbt@gmail.com (R.A.B.); mladinescu@umft.ro (O.F.-M.); 2 Clinical Hospital of Infectious Diseases and Pulmonology Victor Babes Timisoara, Gheorghe Adam Street 13, 300310 Timisoara, Romania; 3 Advanced Instrumental Screening Center, Faculty of Pharmacy, Victor Babes University of Medicine and Pharmacy, Eftimie Murgu Sq. No. 2, 300041 Timisoara, Romania; amalia.ridichie@umft.ro

**Keywords:** pulmonary embolism, pulmonary hypertension, treatment, survival, thrombosis

## Abstract

**Background/Objectives**: Chronic thromboembolic pulmonary hypertension (CTEPH) is characterized by a multitude of underlying causes, treatment modalities and prognostic outcomes. Our aim was to evaluate the underlying causes, comorbidities and survival rates of CTEPH patients. **Methods**: A retrospective analysis was conducted regarding the evolution of CTEPH patients confirmed by right heart catheterization under treatment with specific vasodilator medication in our centre between 2008 and 2023. **Results**: We treated 14 CTEPH patients, 78.57% female, 52.79 ± 13.64 years at inclusion, representing 11.29% of our pulmonary arterial hypertension registry. Initially, the distribution of patients’ NYHA class was II-14.28%, III-71.42% and IV-14.28%. In total, 71.42% of these patients were technically operable due to the central location of the thrombus, but 42.85% presented severe comorbidities and 28.57% refused the surgery or it was financially inaccessible. Only four patients were operated on by pulmonary endarterectomy (PEA). Unfortunately, all the post-PEA patients had persistent pulmonary hypertension and had to continue vasodilator treatment. Overall, 64.28% of patients had monotherapy, 21.42% double therapy and 14.28% triple therapy. Regarding underlying causes and comorbidities, we found the following incidences: 78.57% chronic venous insufficiency, 42.85% obesity, 35.71% thyroid disease, hypertension and hyperuricemia, 21.42% thrombophilia and ischemic heart disease, 14.28% atrial fibrillation, vasculitis and lung disease, and 14.28% neoplastic history and diabetes. Seven patients died (50%), six of whom were unoperated and one of whom was lost (abandoned the program). The survival rates at 1, 3, 5 and 7 years for unoperated patients were 100%, 58.3%, 29.2% and 29.2% versus 100%, 75%, 75% and 75% in post-PEA patients. **Conclusions**: CTEPH, marked by delayed diagnosis, multiple comorbidities and limited intervention options, requires proactive screening and comprehensive multimodal therapies, including PEA, to improve survival rates.

## 1. Introduction

Chronic thromboembolic pulmonary hypertension (CTEPH) manifests as a rare and severe progressive pulmonary vascular disorder, affecting approximately 2–4% of patients post pulmonary embolism (PE) [1]. Alternate etiological pathways may involve smaller and recurrent “silent” pulmonary emboli or in situ thrombosis [2]. Even though CTEPH is often considered a complication of symptomatic acute PE, only 50%–75% of patients exhibit a documented history of acute PE [3,4]. Currently classified as a precapillary form of pulmonary hypertension, CTEPH falls within Class IV of the prevailing 2022 European Society of Cardiology (ESC) and European Respiratory Society (ERS) guidelines for PH [5]. CTEPH diagnosis must be established based on three main criteria: the manifestation of nonspecific clinical symptoms (exertional dyspnoea, fatigue, syncope), detection of residual thrombotic lesions within the lungs and the confirmation of precapillary PH through right heart catheterization (RHC) [6]. The current ESC/ERS diagnostic criteria for CTEPH require the presence of at least one segmental perfusion defect (identified by perfusion scintigraphy or pulmonary arterial obstruction identified by computed tomography, magnetic resonance imaging or conventional angiography), alongside a minimum of three months of effective anticoagulant therapy and the confirmation of precapillary hypertension characterized by mean pulmonary arterial pressure (mPAP) exceeding 20 mmHg, pulmonary artery wedge pressure (PAWP) below or equal to 15 mmHg and pulmonary vascular resistance (PVR) surpassing 2 Wood units (WU) [5]. Additionally, there exist instance of patients diagnosed with chronic thromboembolic pulmonary disease (CTEPD) without accompanying PH; however, these patients constitute a minority within the cohort referred to specialized CTEPH centres [7].

The initial academic reports of CTEPH were documented in 1950, when a few autopsy cases highlighted the presence of thrombi in the pulmonary arteries, which served as the primary diagnostic method at the time [8]. Thirteen years subsequent to these initial reports, the inaugural form of the pulmonary endarterectomy (PEA) procedure was successfully performed [9]. Over the ensuing decades, this therapeutic intervention evolved into the contemporary multimodal approach utilized today, characterized by reduced mortality rates and increased efficacy in thrombus extraction [5,10]. As such, the surgical PEA approach represents the preferred treatment modality for patients with accessible pulmonary arterial lesions [11], primarily due to the fact that this intervention has the potential to result in a normalization of pulmonary haemodynamics (65% decrease in PVR) and functional capacity [12].

In recent years, the number of patients diagnosed with CTEPH has seen a consistent increase, a trend most likely attributable to enhanced comprehension of the disease and improved accessibility to diagnostic investigations [13]. In light of these developments, there has been a concerted effort in numerous countries to introduce increasingly proactive screening initiatives aimed at identifying CTEPH amongst patients who continue to experience persistent dyspnoea subsequent to PE, as well as those individuals who possess risk factors associated with the development of CTEPH [14]. Such is the case of our centre, established in 2008 as part of the National Program for Pulmonary Arterial Hypertension (PAH) in Romania. Although the centre’s initial focus did not explicitly include CTEPH, the increasing recognition of its significance in international guidelines prompted a shift in our centre’s focus towards this PAH variant.

Data concerning the incidence and prevalence of CTEPH often exhibit disparities owing to the heterogeneity in the studied populations, variations in imaging modalities employed and discrepancies in diagnostic criteria as well as the duration of follow-up periods. Notably, estimates of the annual incidence of CTEPH have been reported to range from 3 to 5 cases per 100,000 individuals per year in certain studies [15]. However, alternative registry data indicate a wider range of incidence rates, spanning from 2 to 6 cases per million adults annually, accompanied by corresponding prevalence estimates ranging between 26 and 38 cases per million adults [16,17,18]. Additional data from the United States of America indicate that the incidence of CTEPH subsequent to the initial episode of PE varies between 0.1% and 9.1% [15], while a meta-analysis from 2017 has shown that approximately 0.56% of all patients with a prior diagnosis of PE have CTEPH [19]. A more recent meta-analysis, conducted in 2023 and encompassing 10,249 patients with PE, suggest that the incidence of CTEPH amongst survivors of acute PE would be around 2.7% [11]. In subjects with a prior history of PE, emerging cases of CTEPH typically arise within the initial two years following the PE episode [14].

The onset of CTEPH exhibits considerable variability in age, typically manifesting in adulthood, with an average age of onset reported at 67.5 years, with a slightly higher prevalence in women compared to men [20]. Early detection and precise diagnosis of CTEPH pose varied challenges and may take up to an average of 14 months from the onset of symptoms to diagnosis in expert centres [21,22].

The primary objective of this study was to determine the survival rate among CTEPH patients treated at our regional centre between 2008 and 2023, while concurrently providing a comprehensive analysis of the risk factors, associated comorbidities and the natural progression of the disease. This encompassed an examination of both patients who underwent surgical intervention and those who did not, aiming to offer a general understanding of the clinical course and outcomes associated with CTEPH management in our setting.

## 2. Materials and Methods

This study is grounded in a retrospective observational cohort comprising all patients treated for CTEPH within the Pulmonology Department of the Clinical Hospital of Infectious Diseases and Pulmonology “Victor Babes” Timisoara, as part of the National Program for Pulmonary Arterial Hypertension. The study period extended from 1 January 2008, which marks the establishment of the centre, to 31 December 2023. Patient inclusion in the national program, and consequently in this study, fluctuated over the years in accordance with the ESC/ERS guidelines applicable at the time of enrolment. All patients underwent confirmation of CTEPH via CT pulmonary angiogram (with a small subset also undergoing pulmonary angiography) and precapillary PAH confirmed via RHC. Additionally, patients underwent comprehensive interdisciplinary evaluation using standard methods such as echocardiography, plethysmography, lung diffusion testing (DLCO), Doppler echocardiography, D-Dimers, and six-minute walk test (6MWT), among others. All patients that underwent PEA had a follow-up RHC performed within six months to assess their status postintervention. Each patient’s observation period commenced at the initiation of vasodilator treatment for CTEPH and extended until the occurrence of death or dropout, or until the data cutoff date of 31 December 2023. This involved a detailed investigation into various factors contributing to the development and progression of CTEPH, including but not limited to demographic characteristics, medical history, predisposing conditions and the trajectory of the disease over time.

Due to the retrospective nature and anonymized status of the data employed, individual patient informed consent was not deemed necessary. Approval for the study was obtained from the local hospital research ethics committee.

The categorical variables were described using frequency counts and corresponding percentages, while numerical variables were presented as means with standard deviations, regardless of their distribution. Rank variables with fewer than four values were treated as categorical. Comparisons between groups were executed utilizing both parametric and nonparametric tests, as deemed appropriate. The number of patients with available data (n) was used as the denominator in the calculation of summary statistics. Statistical significance was conducted at a threshold of *p* < 0.05 (95% level of confidence). Kaplan–Meier overall survival probability estimates from time of inclusion were used to perform survival analyses for the two group. Binary logistic regression analysis was employed to compare the two groups with the dependent variable being whether they died. Data analysis was conducted using the Medcalc statistical software (v22.016, Ostend, Belgium).

## 3. Results

In our centre, we provided treatment to a total of 14 patients diagnosed with CTEPH, with the majority being female (78.57%). The mean age of patients at the time of inclusion was 52.78 years, with a standard deviation of 13.64 years, indicating a relatively wide age range within the cohort. Notably, these CTEPH patients accounted for approximately 11.29% of the total entries (124 patients) within our pulmonary arterial hypertension registry. All our patients originate from six counties in western Romania, with an approximate population of 2.5 million people [23], which fall under the purview of our centre.

**Figure 1:**
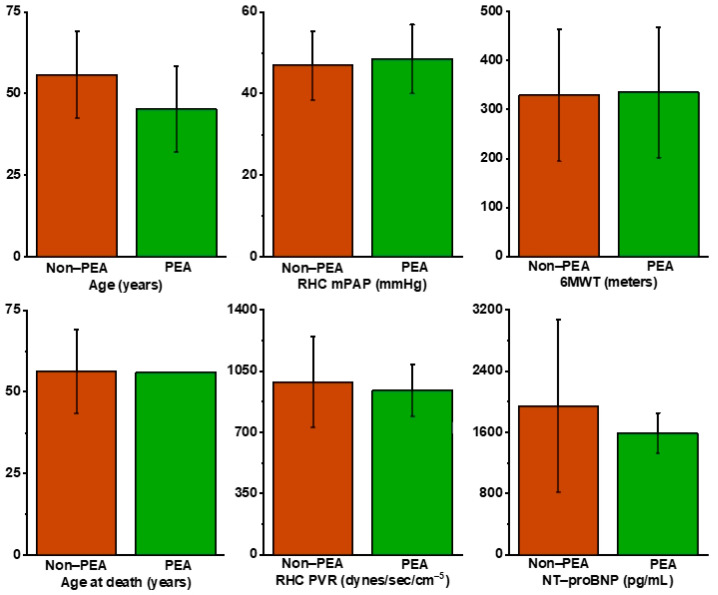
Clustered column chart that visually represents the difference in numerical values between the two groups of CTEPH patients: those who underwent PEA and those who did not.

The CTEPH diagnosis was confirmed following two distinct approaches in our cohort. Firstly, in 64.28% of cases (9 patients), chronic pulmonary embolism was confirmed, which later progressed to associate PAH. Secondly, in 34.72% of cases (5 patients), PAH was initially confirmed and subsequent identification of PE occurred, either as the underlying cause or as a complication. This ‘dual’ presentation underscores the complexity and variability in the diagnostic pathway of CTEPH and highlights the importance of comprehensive evaluation in these patients. Prior to the initiation of the vasodilator treatment program, the distribution of patients according to the New York Heart Association (NYHA) functional class was as follows: 14.28% were classified as NYHA class II, 71.42% as NYHA class III and 14.28% as NYHA class IV. While 71.42% of these patients were technically eligible for surgery due to the central location of the thrombus, a significant proportion, comprising 42.85%, presented with severe comorbidities that contraindicated the intervention. Furthermore, an additional 28.57% declined surgery, either due to financial constraints or logistical barriers. It is noteworthy that five new patients were diagnosed during the COVID-19 pandemic period, an aspect that had influenced treatment decisions and accessibility to healthcare services. In total, only four patients underwent PEA, albeit at a later stage, after an average period of 18.25 months from the time of diagnosis. Regrettably, despite the surgical intervention, all the patients continued to experience persistent pulmonary hypertension post-PEA, requiring ongoing vasodilator treatment. Amongst all of our CTEPH patients, 64.28% received monotherapy, 21.42% double therapy and 14.28% triple therapy. The majority of patients receiving monotherapy were notably prescribed Riociguat. Regarding double therapy, most patients were receiving a combination of Riociguat and Macitentan. Finally, all patients undergoing triple therapy had Treprostinil added to their existing regimen of Riociguat and Macitentan. Notably, the adoption of triple therapy, particularly in the last five years, grew considerably with the availability of prostacyclin analogues in our centre. Another noteworthy aspect regarding patients’ prescribed regimens containing Sildenafil and Bosentan is that these off-label treatment plans were initiated before clear guidelines for CTEPH treatment were established and before the introduction of Riociguat and Treprostinil. Subsequent to the diagnosis of CTEPH, all the patients were initiated on anticoagulant therapy, with the majority (85.71%) prescribed vitamin K antagonists (VKAs). Conversely, a smaller proportion of patients (14.29%) received new oral anticoagulants (NOACs). Table 1 provides a comprehensive breakdown of the data for each individual group as well as the whole cohort. Furthermore, Figure 1 showcases the disparity of the numerical data between the two groups.

In terms of underlying causes and comorbidities, our findings revealed the following distributions within the CTEPH patient population: 78.57% had chronic venous insufficiency, almost three-quarters were either overweight (28.57%) or obese (42.85%), and 35.71% had thyroid disease, hypertension and hyperuricemia. Additionally, 21.42% presented with thrombophilia and ischemic heart disease, while 14.28% had atrial fibrillation, vasculitis and lung disease, as well as a history of neoplastic disease and diabetes.

**Figure 2:**
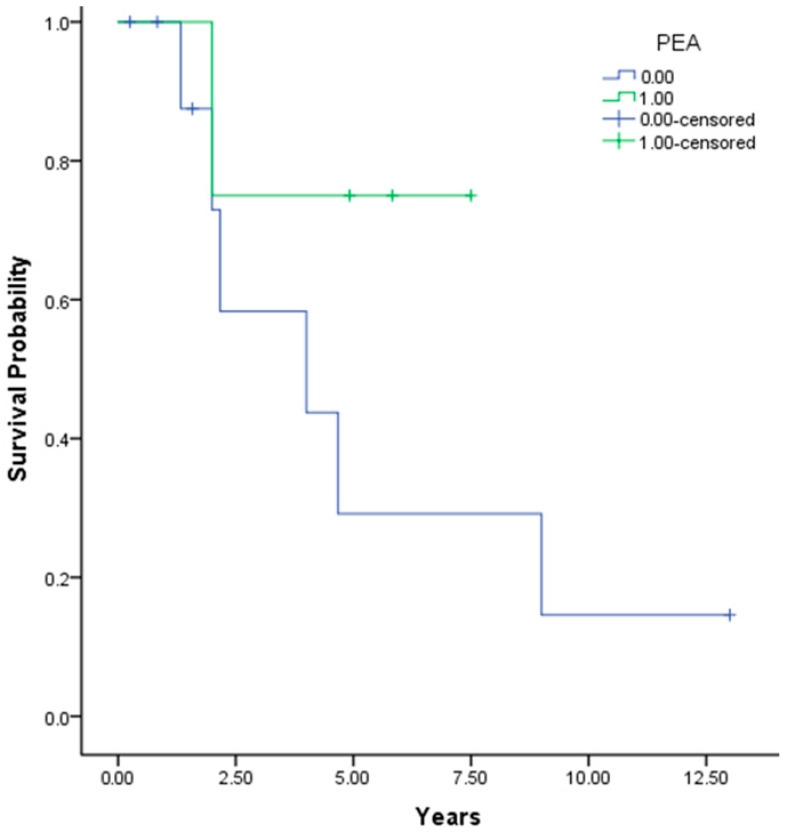
Kaplan–Meier overall survival probability plot, where 1 represents patients who have undergone the intervention.

Out of the cohort, seven patients, constituting 50%, deceased during the study period, with six of these being unoperated and one patient was lost to follow-up (abandoned the program). The overall mean survival time (95% CI) was approximately 6.51 (3.71; 9.30) ± 1.42 years, with a median (95% CI) of 4.67 (0.78; 9.26) ± 2.34 years. Specifically, patients who underwent PEA exhibited a mean survival time (95% CI) of 6.12 (3.79; 8.45) ± 1.19 years, whereas those who had not undergone PEA had a mean survival time (95% CI) of 5.24 (2.28; 8.20) ± 1.50 years. Regarding survival probability, no deaths were recorded within the first year for either group. For patients who did not undergo PEA, the survival probability at 3 years is estimated at 58.3%, at 5 and 7 years at 29.2%, and beyond 9 years at 14.6%. In contrast, for the group that underwent PEA, the survival probability drops to 75% around the 2-year mark and remains consistent at 3, 5 and 7 years, with no additional recorded events (deaths). For a visual representation of this data, please refer to Figure 2.

A Chi-Square statistic test was employed in order to evaluate whether there are statistically significant differences in survival distributions between patients who underwent PEA and those who did not. The Chi-Square value is 1.498, with 1 degree of freedom associated and a *p*-value of 0.221. Furthermore, a binary logistic regression analysis was performed on the two groups with the dependent variable being those who died. The obtained odds ratio for PEA is 0.222, indicating that patients who underwent PEA have approximately 0.222 times the odds of death compared to those who did not undergo PEA. However, this result does not appear to be statistically significant given the associated *p*-value of 0.256.

## 4. Discussion

The majority of our patients were female and presented with a severe form of the disease at the time of diagnosis, with approximately 86% classified as being in functional class NYHA III or IV. This observation aligns with findings from other studies from Europe, such as those conducted in the Czech Republic, which reported similar proportions of patients with an advanced form of the disease [24]. Notably, we observed significant differences in patient age compared to other registries. Our patients were notably younger, with a mean age of 52.78 ± 13.64 years, compared to the reported mean age of 65.2 years in the Czech registry [24] or the 69 ± 13 years in COMPERA registry [25]. This discrepancy may be attributed to underdiagnosis in elderly populations in Romania, highlighting the need for improved awareness and diagnostic strategies in this demographic.

The incidence of CTEPH showed notable variations over the 16 years of the centre’s activity. At the inception of the national program, we were observing approximately one new case every couple of years, with the time gap between the first and second cases spanning roughly four years, however, recent advancements in technological capabilities and the growing focus on this pathophysiological form of PAH (especially after the COVID-19 pandemic) have significantly improved detection rates. As such, in the last few years, we have observed up to 4 new cases confirmed annually (approximately 1.6 cases per million). Given that CTEPH now ranks as the third most prevalent form of PAH in our patient cohort, following idiopathic and congenital heart disease forms, and coupled with the recent surge in newly identified cases of CTEPH, it underscores the significant impact of this subgroup within our patient population. The estimated prevalence within the geographical area under our care is approximately 2.8 cases per million inhabitants (estimated for 2023).

The study findings underscore the prevalence of numerous associated pathologies in these patients, which either serve as inducing risk factors or complications of CTEPH. Among the most frequent risk factors identified were chronic venous insufficiency, obesity and thrombophilia. Additionally, common comorbidities observed included thyroid disease, hypertension, ischemic heart disease and hyperuricemia. These findings, notably consistent with the results reported in other studies [24,25,26,27], highlight the complex interplay of various systemic conditions in the aetiology and clinical course of CTEPH.

Only 64.28% of the patients had a history of pulmonary embolism, while 21.43% lacked venous insufficiency, suggesting that CTEPH can manifest in the absence of a venous thromboembolism history.

Regrettably, only a limited number of patients underwent PEA in our centre, and these surgeries were conducted at a later stage compared to reports from other centres. Specifically, the median time from diagnosis to PEA in our centre was around 18 months, contrasting starkly with the reported figure of 2.9 months in other expert centres. Furthermore, all the patients who underwent PEA in our centre exhibited residual pulmonary hypertension post-procedure, a notable difference from the 34.1% reported in the Czech expert centre [24].

This study also highlights a clear trend where younger patients, with an average age of 45.25 years, were more likely to undergo surgical intervention compared to their older counterparts, who had an average age of 55.82 years and were predominantly managed conservatively. Notably, concerted financial efforts were directed towards facilitating surgical treatment for the younger patients.

In terms of survival, the data from our centre demonstrate notable differences compared to other national-level registries and international reports. For instance, in the Czech centre, the estimated five-year survival probability (95% CI) was 95.3% (89.9; 97.9) for operated patients without residual PH, 86.3% (75.3; 92.7) for operated patients with residual PH and 61.2% (54.0; 67.6) for non-operated patients [24]. Meanwhile, according to the analysis for the entire baseline cohort of COMPERA, survival rates at 1, 2, 3, 4 and 5 years were estimated at 92.0%, 83.9%, 74.7%, 68.3% and 59.8%, respectively [25].

While our patients who underwent PEA had a relatively similar survival rate compared to COMPERA in the first 3 years, we observed a better outcome (75% vs. 68%) in favour of our patients at 4 years and beyond. However, it was considerably lower than the survival rate reported in the Czech register. Conversely, the survival rates for the non-PEA group are remarkably lower than those documented in international datasets. This discrepancy of survival outcomes can be attributed to several factors. Firstly, the predominant use of monotherapy in our patient cohort stems from the limited availability of modern specific medications, particularly prostacyclin analogues, which were only accessible through clinical trials until 2023. Consequently, many patients had limited options and relied solely on monotherapy for a significant duration. With the recent inclusion of these modern molecules, the target became the use of double and triple combinations of medications, indicating a shift towards more aggressive management approaches with hopes of better survival outcomes [28].

A significant challenge in the management of CTEPH in many countries stems from the absence of dedicated centres and comprehensive epidemiological data on the incidence and prevalence of this condition. Consequently, cases often remain undiagnosed until they reach an advanced stage, leading to limited therapeutic options. Despite being considered the gold standard treatment for CTEPH, PEA is not currently performed in Romania. Only sporadic cases of PEA have been conducted through collaboration with experts from dedicated European centres. As a result, most patients requiring PEA undergo surgery abroad, incurring high financial costs and causing significant psychological and emotional distress for both patients and their families.

While PEA stands as the main curative treatment option for cases where organized thrombi are situated in the central part of the pulmonary artery, effective treatment options have historically been lacking for cases where thrombi are located in the peripheral part of the pulmonary artery. In recent years, BPA, a transcatheter procedure designed to dilate stenotic or occluded lesions in the peripheral pulmonary artery, has seen multiple developments. If initially BPA was at one point abandoned due to concerns regarding haemorrhagic complications, Japanese experts have made significant advancements in the technique, leading to improvements in its safety and efficacy. Consequently, BPA is now being reevaluated worldwide as a viable treatment option for CTEPH [29,30]. The BPA procedure was introduced in Romania as recently as 2023. Consequently, drug therapy remained the primary treatment option for patients with CTEPH until that time.

Initially, medical therapies were employed off-label, relying on uncontrolled studies and/or regional approvals. However, the landscape has since evolved, with three successful randomized clinical trials conducted. Consequently, Riociguat, belonging to a novel class of compounds known as soluble guanylate cyclase stimulators, stands as the first approved medication for the management of CTEPH [31]. Furthermore, the use of Treprostinil, a prostacyclin analogue [32] and/or Macitentan, an endothelin receptor antagonist [33] in patients with inoperable CTEPH or those with persistent/recurrent PH after PEA, proved to be effective. In terms of vasodilator medication for the treatment of CTEPH, Riociguat has been available in Romania since 2015 and was approved for use at a European level in 2014 [34,35]. Other treatment options available for CTEPH management included Macitentan. Furthermore, starting from 2023, Treprostinil with continuous subcutaneous administration via pump became another therapeutic option for CTEPH patients. Fortunately, some patients have been able to benefit from compensation programs and access medications through participation in clinical trials that involved our centre.

General measures recommended for all our patients with CTEPH include lifelong therapeutic anticoagulation, utilizing VKAs or NOACs. This approach is vital due to the presence of recurrent pulmonary thromboembolism and inadequate clot resolution, which are key pathophysiological features of the disease. It is important to note that particular attention is warranted for patients with antiphospholipid syndrome, which according to some studies constitutes approximately 10% of the CTEPH population, with VKAs being the recommended choice for anticoagulation in such cases [36,37,38]. Diuretics and oxygen therapy were also recommended as adjunctive treatments, depending on individual patient needs. Additionally, supervised exercise training, which has been shown to be effective and safe, particularly in inoperable CTEPH patients and in the early post-PEA period [39,40] has been performed.

The study’s limitations include the relatively small number of patients, especially when compared to larger European or American registries. Furthermore, the underdiagnosis in elderly populations in Romania, coupled with the fact that patients were diagnosed and treated over an extended period, with evolving diagnostic and therapeutic options, poses a challenge for reporting consistent outcomes. Additionally, the need for patients to undergo PEA at different outside centres, such as Vienna and Pavia, implies that the intervention may not have been uniformly conducted for all patients, depending on the expertise available at each centre. Nevertheless, considering the rarity of CTEPH cases and the extensive duration of patient monitoring in our study, the observations presented retain their significance. They offer valuable insights into the clinical trajectory and management of CTEPH, while also shedding light on the specific context within Romania.

## 5. Conclusions

The rising incidence of CTEPH diagnoses in our centre highlights the urgent need for enhanced management strategies. All our patients presented with a multitude of associated comorbidities and risk factors. Moreover, the diagnosis of CTEPH tends to be established later than in other reports. Compounding this issue is the challenging access to interventions such as PEA; this situation ultimately contributes to poorer survival rates compared to other registries. Indeed, proactive screening of high-risk individuals and the establishment of a dedicated national CTEPH expert centre are crucial steps toward enhancing the management of this severe pathology. Finally, expediting the diagnosis process, adopting a multimodal therapeutic approach and, in particular, reducing the delay to PEA could substantially improve the survival outcomes for individuals with CTEPH.

## Figures and Tables

**Table 1:** Descriptive statistics of numerical and categorical data from our centre.

Parameter	All CTEPH Patients n = 14	Non-Operated Patients n = 10	PEA-Operated Patients n = 4
Age (years) ^(a)^	52.79 ± 13.64	55.80 ± 13.25	45.25 ± 13.17
Gender (female) ^(b)^	11 (78.57%)	8 (80%)	3 (75%)
NYHA I & II ^(b)^	2 (14.28%)	1 (10%)	1 (25%)
NYHA III ^(b)^	10 (71.42%)	7 (70%)	3 (75%)
NYHA IV ^(b)^	2 (14.28%)	2 (20%)	-
Deaths ^(b)^	7 (50%)	6 (60%)	1 (25%)
Age at death (years) ^(a)^	56.14 ± 11.90	56.16 ± 12.86	56.00
6MWT (meters) ^(a)^	330.64 ± 134.21	328.90 ± 134.73	335.00 ± 132.90
Duration of vasodilator treatment (years) ^(a)^	4.22 ± 3.62	3.88 ± 3.88	5.06 ± 1.99
NT-proBNP (pg/mL) ^(a)^	1838.07 ± 1011.44	1938.70 ± 1125.71	1586.5 ± 260.91
RHC mPAP (mmHg) ^(a)^	47.36 ± 8.78	46.90 ± 8.41	48.50 ± 8.44
RHC PVR (dynes/sec/cm^−5^) ^(a)^	972.80 ± 243.20	986.24 ± 259.76	940.00 ± 149.76
PE history ^(b)^	9 (64.28%)	5 (50%)	4 (100%)
DVT history ^(b)^	11 (78.57%)	8 (80%)	3 (75%)
Obesity ^(b)^	6 (42.85%)	4 (40%)	2 (50%)
Thrombophilia ^(b)^	3 (21.42%)	1 (10%)	2 (50%)
VKAs ^(b)^	12 (85.71%)	8 (80%)	4 (100%)
NOACs ^(b)^	2 (14.28%)	2 (20%)	-
Monotherapy ^(b)^	9 (64.28%)	8 (80%)	1 (25%)
Double therapy ^(b)^	3 (21.42%)	2 (20%)	1 (25%)
Triple therapy ^(b)^	2 (14.28%)	-	2 (50%)

NYHA, New York Heart Association; 6MWT, six-minute walking test; NT-proBNP, N-terminal prohormone of brain natriuretic peptide; RHC, right heart catheterization; mPAP, mean pulmonary arterial pressure; PVR, pulmonary vascular resistance; PE, pulmonary embolism; DVT, deep vein thrombosis; VKAs, vitamin K antagonists; NOACs, new oral anticoagulants. ^(a)^ Mean ± standard deviation. ^(b)^ Observed frequencies in percentages.

## Data Availability

The datasets are not publicly available. Anonymized data may be provided upon request from Razvan Adrian Bertici.

## References

[1] Delcroix M., Kerr K., Fedullo P. (2016). Chronic Thromboembolic Pulmonary Hypertension. Epidemiology and Risk Factors. Ann. Am. Thorac. Soc..

[2] Egermayer P., Peacock A.J. (2000). Is pulmonary embolism a common cause of chronic pulmonary hypertension? Limitations of the embolic hypothesis. Eur. Respir. J..

[3] Pepke-Zaba J., Delcroix M., Lang I., Mayer E., Jansa P., Ambroz D., Treacy C., D’Armini A.M., Morsolini M., Snijder R. (2011). Chronic Thromboembolic Pulmonary Hypertension (CTEPH). Circulation.

[4] Lang I.M., Madani M. (2014). Update on Chronic Thromboembolic Pulmonary Hypertension. Circulation.

[5] Humbert M., Kovacs G., Hoeper M.M., Badagliacca R., Berger R.M.F., Brida M., Carlsen J., Coats A.J.S., Escribano-Subias P., Ferrari P. (2023). 2022 ESC/ERS Guidelines for the diagnosis and treatment of pulmonary hypertension. Eur. Respir. J..

[6] Gopalan D., Delcroix M., Held M. (2017). Diagnosis of chronic thromboembolic pulmonary hypertension. Eur. Respir. Rev..

[7] Swietlik E., Ruggiero A., Fletcher A.J., Taboada D., Knightbridge E., Harlow L., Harvey I., Screaton N., Cannon J.E., Sheares K.K. (2019). Limitations of resting haemodynamics in chronic thromboembolic disease without pulmonary hypertension. Eur. Respir. J..

[8] Carroll D. (1950). Chronic obstruction of major pulmonary arteries. Am. J. Med..

[9] Houk V.N., Hufnagel C.A., McClenathan J.E., Moser K.M. (1963). Chronic thrombotic obstruction of major pulmonary arteries. Am. J. Med..

[10] Ghofrani H.A., D’Armini A.M., Kim N.H., Mayer E., Simonneau G. (2021). Interventional and pharmacological management of chronic thromboembolic pulmonary hypertension. Respir. Med..

[11] Luijten D., Talerico R., Barco S., Cannegieter S.C., Delcroix M., Ende-Verhaar Y.M., Huisman M.V., Konstantinidis S., Mairuhu A.T., van Mens T.E. (2023). Incidence of chronic thromboembolic pulmonary hypertension after acute pulmonary embolism: An updated systematic review and meta-analysis. Eur. Respir. J..

[12] Hsieh W.C., Jansa P., Huang W.C., Nižnanský M., Omara M., Lindner J. (2018). Residual pulmonary hypertension after pulmonary endarterectomy: A meta-analysis. J. Thorac. Cardiovasc. Surg..

[13] Klok F.A., Couturaud F., Delcroix M., Humbert M. (2020). Diagnosis of chronic thromboembolic pulmonary hypertension after acute pulmonary embolism. Eur. Respir. J..

[14] Klok F.A., van Kralingen K.W., van Dijk A.P.J., Heyning F.H., Vliegen H.W., Huisman M.V. (2010). Prospective cardiopulmonary screening program to detect chronic thromboembolic pulmonary hypertension in patients after acute pulmonary embolism. Haematologica.

[15] Gall H., Hoeper M.M., Richter M.J., Cacheris W., Hinzmann B., Mayer E. (2017). An epidemiological analysis of the burden of chronic thromboembolic pulmonary hypertension in the USA, Europe and Japan. Eur. Respir. Rev..

[16] Leber L., Beaudet A., Muller A. (2021). Epidemiology of pulmonary arterial hypertension and chronic thromboembolic pulmonary hypertension: Identification of the most accurate estimates from a systematic literature review. Pulm. Circ..

[17] Delcroix M., Torbicki A., Gopalan D., Sitbon O., Klok F.A., Lang I., Jenkins D., Kim N.H., Humbert M., Jais X. (2021). ERS statement on chronic thromboembolic pulmonary hypertension. Eur. Respir. J..

[18] Kramm T., Wilkens H., Fuge J., Schäfers H.-J., Guth S., Wiedenroth C.B., Weingard B., Huscher D., Pittrow D., Cebotari S. (2018). Incidence and characteristics of chronic thromboembolic pulmonary hypertension in Germany. Clin. Res. Cardiol..

[19] Ende-Verhaar Y.M., Cannegieter S.C., Noordegraaf A.V., Delcroix M., Pruszczyk P., Mairuhu A.T.A., Huisman M.V., Klok F.A. (2017). Incidence of chronic thromboembolic pulmonary hypertension after acute pulmonary embolism: A contemporary view of the published literature. Eur. Respir. J..

[20] Schweikert B., Pittrow D., Vizza C.D., Pepke-Zaba J., Hoeper M.M., Gabriel A., Berg J., Sikirica M. (2014). Demographics, clinical characteristics, health resource utilization and cost of chronic thromboembolic pulmonary hypertension patients: Retrospective results from six European countries. BMC Health Serv. Res..

[21] Klok F.A., Barco S., Konstantinides S.V., Dartevelle P., Fadel E., Jenkins D., Kim N.H., Madani M., Matsubara H., Mayer E. (2018). Determinants of diagnostic delay in chronic thromboembolic pulmonary hypertension: Results from the European CTEPH Registry. Eur. Respir. J..

[22] Kopeć G., Forfia P., Abe K., Beaudet A., Gressin V., Jevnikar M., Meijer C., Tan Y.Z., Moiseeva O., Sheares K. (2024). Recognition, diagnosis, and operability assessment of chronic thromboembolic pulmonary hypertension (CTEPH): A global cross-sectional scientific survey (CLARITY). Pulm. Circ..

[23] National Institute of Statistics (Romania) (2022). Population and Housing Census, 2021—Provisional Results [Internet]. https://insse.ro/cms/en/content/population-and-housing-census-2021-provisional-results.

[24] Jansa P., Ambrož D., Kuhn M., Dytrych V., Aschermann M., Černý V., Gressin V., Heller S., Kunstýř J., Širanec M. (2022). Epidemiology of chronic thromboembolic pulmonary hypertension (CTEPH) in the Czech Republic. Pulm. Circ..

[25] Delcroix M., Staehler G., Gall H., Grünig E., Held M., Halank M., Klose H., Vonk-Noordegraaf A., Rosenkranz S., Pepke-Zaba J. (2018). Risk assessment in medically treated chronic thromboembolic pulmonary hypertension patients. Eur. Respir. J..

[26] Dardi F., Manes A., Guarino D., Suarez S.M., Loforte A., Rotunno M., Pacini D., Galiè N., Palazzini M. (2022). Long-term outcomes after pulmonary endarterectomy. Ann. Cardiothorac. Surg..

[27] Ruaro B., Baratella E., Caforio G., Confalonieri P., Wade B., Marrocchio C., Geri P., Pozzan R., Andrisano A.G., Cova M.A. (2022). Chronic Thromboembolic Pulmonary Hypertension: An Update. Diagnostics.

[28] Ribas Sola J., Sánchez-Corral Mena M.Á., Riera-Mestre A. (2024). Update in the management of chronic thrombo-embolic pulmonary hypertension. Med. Clín. (Engl. Ed.).

[29] Nakazato K., Sugimoto K., Oikawa M., Takeishi Y. (2023). Balloon pulmonary angioplasty for chronic thromboembolic pulmonary hypertension:its history and development, and regional medical cooperation in Fukushima. Fukushima J. Med. Sci..

[30] Darocha S., Roik M., Kopeć G., Araszkiewicz A., Furdal M., Lewandowski M., Jacheć W., Grabka M., Banaszkiewicz M., Pietrasik A. (2022). Balloon pulmonary angioplasty in chronic thromboembolic pulmonary hypertension: A multicentre registry. EuroIntervention.

[31] Ghofrani H.A., D’Armini A.M., Grimminger F., Hoeper M.M., Jansa P., Kim N.H., Mayer E., Simonneau G., Wilkins M.R., Fritsch A. (2013). Riociguat for the Treatment of Chronic Thromboembolic Pulmonary Hypertension. N. Engl. J. Med..

[32] Sadushi-Kolici R., Jansa P., Kopec G., Torbicki A., Skoro-Sajer N., Campean I.-A., Halank M., Simkova I., Karlocai K., Steringer-Mascherbauer R. (2019). Subcutaneous treprostinil for the treatment of severe non-operable chronic thromboembolic pulmonary hypertension (CTREPH): A double-blind, phase 3, randomised controlled trial. Lancet Respir. Med..

[33] Ghofrani H.A., Simonneau G., D’Armini A.M., Fedullo P., Howard L.S., Jaïs X., Jenkins D.P., Jing Z.-C., Madani M.M., Martin N. (2017). Macitentan for the treatment of inoperable chronic thromboembolic pulmonary hypertension (MERIT-1): Results from the multicentre, phase 2, randomised, double-blind, placebo-controlled study. Lancet Respir. Med..

[34] Kim N.H., Mayer E. (2015). Chronic thromboembolic pulmonary hypertension: The evolving treatment landscape. Eur. Respir. Rev..

[35] Stasch J.P., Pacher P., Evgenov O.V. (2011). Soluble Guanylate Cyclase as an Emerging Therapeutic Target in Cardiopulmonary Disease. Circulation.

[36] Ordi-Ros J., Sáez-Comet L., Pérez-Conesa M., Vidal X., Riera-Mestre A., Castro-Salomó A., Cuquet-Pedragosa J., Ortiz-Santamaria V., Mauri-Plana M., Solé C. (2019). Rivaroxaban Versus Vitamin K Antagonist in Antiphospholipid Syndrome. Ann. Intern. Med..

[37] Pengo V., Denas G., Zoppellaro G., Jose S.P., Hoxha A., Ruffatti A., Andreoli L., Tincani A., Cenci C., Prisco D. (2018). Rivaroxaban vs warfarin in high-risk patients with antiphospholipid syndrome. Blood.

[38] Konstantinides S.V., Meyer G., Becattini C., Bueno H., Geersing G.J., Harjola V.P., Huisman M.V., Humbert M., Jennings C.S., Jiménez D. (2020). 2019 ESC Guidelines for the diagnosis and management of acute pulmonary embolism developed in collaboration with the European Respiratory Society (ERS). Eur. Heart J..

[39] Nagel C., Prange F., Guth S., Herb J., Ehlken N., Fischer C., Reichenberger F., Rosenkranz S., Seyfarth H.-J., Mayer E. (2012). Exercise Training Improves Exercise Capacity and Quality of Life in Patients with Inoperable or Residual Chronic Thromboembolic Pulmonary Hypertension. PLoS ONE.

[40] Nagel C., Nasereddin M., Benjamin N., Egenlauf B., Harutyunova S., Eichstaedt C.A., Xanthouli P., Mayer E., Grünig E., Guth S. (2020). Supervised Exercise Training in Patients with Chronic Thromboembolic Pulmonary Hypertension as Early Follow-Up Treatment after Pulmonary Endarterectomy: A Prospective Cohort Study. Respiration.

